# Episodic Cancer Pain: Patient Reporting, Prevalence, and Clinicodemographic Associations at Initial Cancer Pain Clinic Assessment

**DOI:** 10.1155/2020/6190862

**Published:** 2020-05-22

**Authors:** Paulo Reis-Pina, Anand Acharya, Antonio Barbosa, Peter G. Lawlor

**Affiliations:** ^1^Palliative Care Unit, Casa de Saúde da Idanha, Sintra, Portugal; ^2^Instituto Português de Oncologia, Lisboa, Portugal; ^3^Department of Economics, Carleton University, Ottawa, Canada; ^4^Department of Psychiatry, Centro Hospitalar Lisboa Norte, Centre of Bioethics & Palliative Care Studies Division, Faculdade de Medicina, Universidade de Lisboa, Lisbon, Portugal; ^5^Bruyère Research Institute, Bruyère Continuing Care, Ottawa Hospital Research Institute, The Ottawa Hospital, Ottawa, ON, Canada; ^6^Division of Palliative Care, Department of Medicine, University of Ottawa, Ottawa, ON, Canada

## Abstract

**Background:**

Better understanding of the episodic cancer pain (CP) spectrum, including pains that occur in addition to its conventionally defined breakthrough CP (BTcP) and incident CP (IcP) components, may inform CP assessment and management. This study aimed to determine the prevalence of episodic patient-reported CP and the prevalence and associations of study-defined BTcP (S-BTcP) and IcP (S-IcP) in patients with CP.

**Methods:**

In a cross-sectional study at their first CP clinic attendance, participants with CP had the following assessments: Brief Pain Inventory (BPI); Pain Management Index (PMI), with PMI-negative status indicating undertreatment; standardized neuropathic pain component (NPC) status; S-BTcP (no trigger identified) and S-IcP (trigger identified) status, based on a preceding 7-day history of transitory pain flares distinct from background pain, and BPI-Worst or BPI-Now pain intensity ≥ 4. Clinicodemographic variables' association with S-BTcP and S-IcP was examined in logistic regression analyses.

**Results:**

Of 371 participants, 308 (83%) had episodic CP by history alone; 140 (37.7%) and 181 (48.8%) had S-BTcP and S-IcP, respectively. Multivariable analyses demonstrated significant (*p* < 0.05) associations (odds ratios: 95% CIs) for 6 variables with S-BTcP: head and neck pain location (2.53; 1.20–5.37), NPC (2.39; 1.34–4.26), BPI average pain (1.64; 1.36–1.99), abdominal pain (0.324; 0.120–0.873), S-IcP (0.207; 0.116–0.369), and PMI-negative status (0.443; 0.213–0.918). Similar independent associations (*p* < 0.05) occurred for S-IcP with NPC, BPI average pain, and PMI-negative status, in addition to radiotherapy, S-BTcP, soft tissue pain, and sleep interference.

**Conclusions:**

Episodic or transient patient-reported CP flares often do not meet the more conventional criteria that define BTcP and IcP, the principal episodic CP types. Both BTcP and IcP occur frequently and both are associated with a NPC, higher pain intensity, and less opioid underuse in the management of CP. Further studies are warranted to both better understand the complex presentations of episodic CP and inform its classification.

## 1. Introduction

The prevalence of cancer pain (CP) ranges from 55% (95% CI, 46–64) during active anticancer treatment to 64% (95% CI, 58–75) in advanced disease [1]. Poorly controlled CP generates distress and impaired quality of life [2, 3]. Undertreatment of CP occurs in one-third of patients [4]. Identification of challenging components of the CP presentation guides targeted therapeutic intervention [5], whereas inadequate assessment and classification lead to undertreatment [6, 7]. Effective CP management therefore requires comprehensive, systematic, and multidimensional assessment. Identifying transitory CP flares or exacerbations, broadly referred to as episodic pain, is an important component of such a multidimensional assessment. Depending on the context, episodic CP is referred to as either breakthrough (BTcP) or incident (IcP) [8–10].

Breakthrough pain was first defined in a 1990 study as “a transitory increase in pain to greater than moderate intensity (that is, to an intensity of “severe” or “excruciating”), which occurred on a baseline pain of moderate intensity or less” [11]. Using pain intensity categories (“none”, “slight”, “moderate”, “severe”, and “excruciating”) and defining baseline pain as the average pain intensity experienced for ≥12 hours during the 24 hours prior to interview, those with “severe” or “excruciating” baseline pain were considered to have uncontrolled baseline pain, and BTcP was not possible in that context. The study included BTcP with apparent end-of-dose (opioid) failure. Subsequently, a task group published largely similar algorithmic diagnostic criteria for BTcP but excluded patients with end-of-dose failure and redesignated pain severity as “none”, “mild”, “moderate,” and “severe” [12]. This algorithm identified 41.5% of patients with spontaneous BTcP (no trigger) and 44% with IcP (a pain trigger was identified) in a large European study [13]. A systematic review of BTcP prevalence included all observational or experimental studies on “transitory pain exacerbation or pain flare”, regardless of specific study definition and reported prevalence of 39.9% and 80.5% for broadly defined BTcP in outpatient clinics and hospice settings, respectively [14].

Less commonly, others have designated “incident pain” as an encompassing term for “episodic increases in pain intensity” and “breakthrough” as a term to describe “break through an existing analgesic regimen” [9]. The IcP term was incorporated into the Edmonton Classification System for Cancer Pain (ECS-CP) [8], which defines it as having a moderate or severe intensity that is greater than background pain, often an identifiable trigger, rapid onset (within 5 minutes), and transient and recurring nature. The term BTcP is most commonly used, but there is no widely accepted definition or classification system for BTcP [15]. Furthermore, the term “breakthrough” translates poorly into some languages, presenting yet another taxonomy challenge [10, 16, 17].

Meanwhile, episodic pain has been proposed as an overarching term, initially by a European Association of Palliative Care (EAPC) expert working group [10] and more recently arising from an EAPC Research Network Delphi survey [18], to encompass both BTcP and IcP, in addition to other transitory exacerbations of CP that do not meet the various definitions of BTcP and IcP. The survey also concluded that transient CP exacerbations can occur irrespective of opioid use, presence of background pain, or its control. Some of these conclusions are ostensibly at variance with earlier published recommendations for the diagnostic criteria of BTcP [11, 12, 19, 20]. Regardless of taxonomy issues with BTcP or IcP [18, 19, 21], studies to date suggest that the presence of BTcP, inclusive of an incident component, is associated with higher pain intensity [17, 22], longer time to stable pain control [23, 24], and compromised health-related quality of life [13, 22, 25].

An improved understanding of the clinical presentations of episodic CP types may help to advance their assessment and management. Using a secondary analysis of data, this study aimed to (1) estimate the prevalence of broader patient-reported episodic CP in addition to the prevalence of study-defined episodic BTcP (S-BTcP) and IcP (S-IcP) in patients attending a CP clinic and (2) determine the association of common clinicodemographic factors with episodic S-BTcP and S-IcP.

## 2. Methods

### 2.1. Study Setting and Design

This cross-sectional study was conducted from June 1^st^ 2009 to April 30^th^ 2010 at patients' first clinic consultation in the specialist outpatient CP clinic of the Portuguese Cancer Institute, a tertiary-level cancer centre in Lisbon, Portugal. The local Research Ethics Board approved the primary cross-sectional collection of data as part of a longitudinal study examining the predictors of time to achieve stable cancer pain control.

### 2.2. Study Participants and Eligibility Criteria

Consecutively referred clinic patients were approached for consent to participate. The inclusion eligibility criteria were s follows: adult patients (>18 years old) with a cancer diagnosis, who provided informed consent to study participation, capable of rating pain intensity on a numerical scale (0, no pain; 10, worst pain imaginable). Patients without active cancer or with non-CP were excluded; CP included pain related directly to malignant involvement or to anticancer treatments.

### 2.3. General Assessment Measures and Data Recording

Patients underwent standardized assessment using Portuguese versions of validated tools, and clinical data were routinely recorded. The CAGE (Cut down, Annoy, Guilt, Eyeopener) alcohol questionnaire screened for a history of alcohol use disorder [26, 27]. The Eastern Cooperative Oncology Group (ECOG) scale was used to rate the functional performance status [28]. Scores ≥ 4 on the Short Portable Mental Status Questionnaire [29], >7 on the anxiety and depression subscales of the Hospital Anxiety and Depression Scale (HADS) [30], and ≥4 on the Emotion Thermometer (ET) tool [31, 32], screened for cognitive impairment, anxiety, depression, and emotional distress, respectively. Primary cancer diagnoses and types of metastases were recorded. The oncology team's documentation of palliative status when present was noted. Chemotherapy, radiotherapy, or surgery administered ≤ 30 days were also recorded.

### 2.4. Cancer Pain Severity and Treatment

Brief Pain Inventory (BPI) pain intensity ratings (worst, average, and least in the last 7 days) and pain now (0–10) were recorded with percentage relief pain (0–100%) [33], principal pain location, and pain duration. The standard oral morphine equivalent daily dose (MEDD) was calculated [34] and recorded along with the type and number of current adjuvant (pharmacological) analgesic treatments.

### 2.5. Cancer Pain Mechanism and Topography

All referred patients had the Douleur Neuropathique 4 (DN4) [35, 36] and neuropathic pain screening tool administered. As previously reported [37], physicians classified the pain mechanism for a patient's main CP, based on history, physical examination, and review of diagnostic imaging, as either solely nociceptive or mixed (nociceptive and neuropathic). The designation of a neuropathic pain component (NPC) was based on a combination of both a DN4 score of ≥4 and a physician's clinical assessment suggesting the presence of a neuropathic mechanistic component. Nociceptive pain was topographically categorized as visceral, bone, and soft tissue in origin.

### 2.6. Study-Defined Episodic Pain

Episodic pain, defined as a transitory flare of pain that occurs either in the absence of or in addition to baseline pain over the course of the preceding 7 days, was primarily recorded on the basis of history; it was further subdivided into “incident pain” when a trigger or incident activity was identifiable by the patient and “breakthrough pain” when no trigger was identified.

Attempting to better align with conventional definitions of BTcP and IcP, we further refined the initial history-based designation of episodic pain by using BPI-worst scores to generate a post hoc study designation of both S-BTcP and S-IcP. The designation of either S-BTcP or S-IcP additionally required patients to have a BPI-worst or BPI-now score ≥ 4. Based on mild (0–3), moderate (4–6), and severe (7–10) pain intensity categories, application of the BPI score criteria meant that patients with either S-BTcP or S-IcP had reported pain in a moderate or severe category.

### 2.7. Data Analyses

Means are expressed with standard deviations, and medians are expressed with the interquartile range (IQR) unless otherwise stated. Continuous and categorical variables reflecting the potential functional and psychosocial impact of episodic pain types and relevant pharmacological interventions underwent group comparisons in relation to presence or absence of S-BTcP and S-IcP, using *t* tests and either chi-squared or Fisher's exact tests, respectively. The Wilcoxon rank-sum test was used for group comparison of initial MEDD values prior to their logarithmic transformation (log_n_MEDD) for further analyses. Subgroup comparisons of the S-BTcP-only and S-IcP-only groups were also conducted in relation to the same set of clinicodemographic variables as used in the larger group comparisons.

Logistic regression was used to test the strength of association of variables with S-BTcP and S-IcP. The independent variable specification for multivariable models was based on clinical plausibility, avoidance of multicollinearity, limits imposed by the frequency of dependent variable outcomes, and having a *p* < 0.25 value in initial bivariable analyses. Independent variables were block-entered into multivariable models with S-BTcP and S-IcP as dependent variables, generating odds ratios (ORs) and 95% confidence intervals (CIs). Indicator (dummy) variables were designated for multilevel categorical variables. Backward elimination was used to select final multivariable models; goodness of fit and discrimination were assessed using the Hosmer–Lemeshow test and the area under the receiver operating characteristic curve (AUC), respectively. Data were analyzed using Stata/IC, V14.2 [38], and statistical significance was set at *p* < 0.05.

## 3. Results

### 3.1. Prevalence of Episodic Pain Types

**Figure 1:**
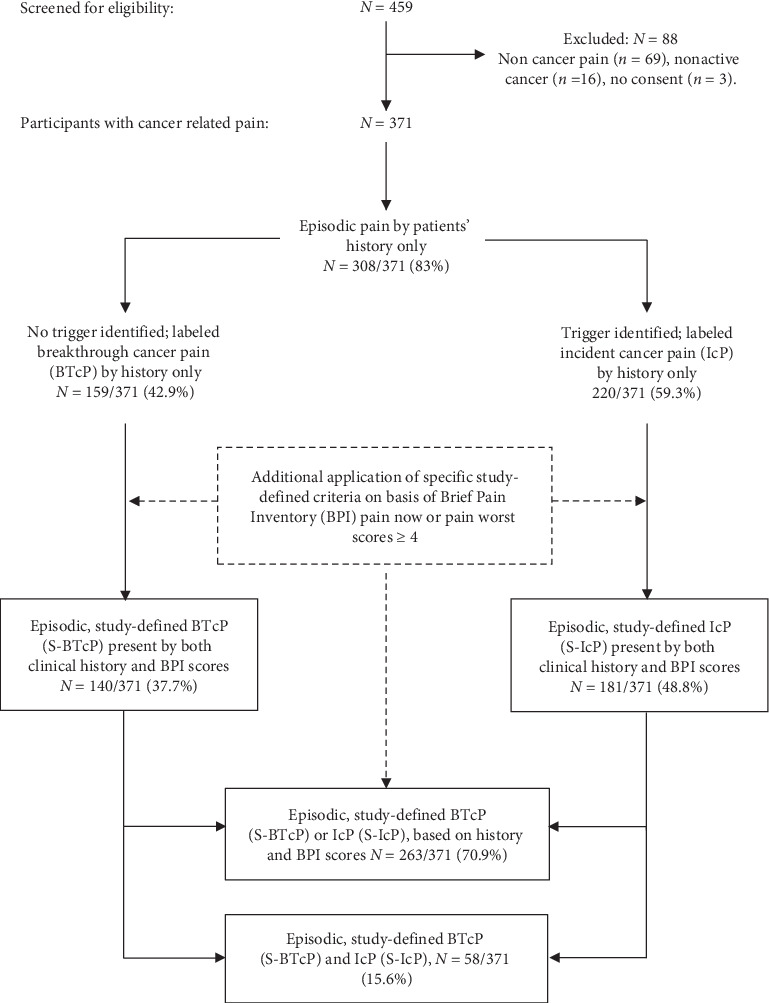
Study flow diagram.

The study sample derivation is illustrated in Figure 1. Of 459 patients, 88 were excluded because of non-CP (*n* = 69), nonactive cancer (*n* = 16), or failure to consent (*n* = 3). Of the study sample (*N* = 371), 63 (17%) patients had no transitory pain, leaving a total of 308 (83%) with episodic pain by history alone. After applying the specific study-defined criteria, there were 140 (37.7%) and 181 (48.8%) patients with S-BTcP and S-IcP, respectively. Of the study sample, 263 (70.9%) had pain of either S-BTcP or S-IcP types; 58 (15.6%) had both types.

### 3.2. General Clinical and Demographic Profiles of the Study Sample and Episodic Pain Groups

Clinical and demographic characteristics of the study sample (*n* = 371) and episodic pain groups are summarized in Table 1. In the study sample, the mean age was 62.1 ± 14.3 years; 199 (54%) were female; 263 (71%) had metastatic disease of at least one site, most commonly bone (34.8%); and 176 (47%) had their oncological treatment goal documented as palliative. There was a high proportion of head and neck cancers (24.8%) and low proportion of lung cancers (2.7%). Most patients (83%) had ECOG scores of 0–2. Of the study sample, 30.2%, 45%, and 47.4% had, respectively, received recent surgery, chemotherapy, and radiotherapy.

Compared with those without breakthrough pain, S-BTcP occurred more frequently (34.3% vs 19.1%) in those with head and neck cancers and less frequently (8.6% vs 13.4%) in those with breast cancer (*p*=0.022). A palliative treatment goal was documented more frequently (56.4% vs 42%) in association with S-BTcP (*p*=0.007). Compared to those without incident pain, metastatic lung and central nervous system metastases occurred more frequently in association with S-IcP (18.2% vs 10.5% and 8.3% vs 3.2%; *p*=0.034 and 0.033, respectively). Recent exposure to chemotherapy and radiotherapy was more frequently associated with S-IcP (50.3% vs 40% and 58.6% vs 36.8%; *p*=0.047 and *p* < 0.001, respectively).

### 3.3. Cancer Pain Data and Psychosocial Assessments in the S-BTcP Group

Participants' CP characteristics according to S-BTcP status are presented in Table 2. S-BTcP was more often associated with a NPC (47.1% vs 23.4%; *p* < 0.001) and soft tissue origin (78.6% vs 46.8%; *p* < 0.001). S-IcP occurred less frequently (41.4% vs 53.3%; *p*=0.027) in the presence of S-BTcP. A head and neck pain location occurred more frequently (38.6% vs 15.6%), and an abdominal pain location less frequently (7.9% vs 16.9%) in those with S-BTcP. S-BTcP was associated with higher “worst” (8.4 ± 1.6 vs 6.7 ± 2.9), “least” (3.6 ± 1.5 vs 2.6 ± 2.0), “average” (5.6 ± 1.4 vs 4.5 ± 2.1), and “now” (6.2 ± 1.9 vs 4.9 ± 2.8) pain ratings and lower percentage pain relief (36.4 ± 26.1 vs 49.7 ± 29.9) in BPI assessments (*p* < 0.0001 for all differences). There were no statistically significant differences in BPI interference scores based on S-BTcP status.

Participants' CP management with opioids and adjuvant analgesics according to S-BTcP status are summarized in Table 3. The MEDD was higher (log_n_MEDD: 3.6 ± 0.9 vs 3.1 ± 1.5, *p*=0.0005) in those with S-BTcP. Absence of a regularly prescribed opioid occurred less frequently in relation to S-BTcP (2.1% vs 16.9%; *p* < 0.001). Antiepileptic use was higher (24.3% vs 15.2%, *p*=0.028) in those with S-BTcP. A substance abuse history occurred more frequently (28.6% vs 19.9%; *p*=0.055) in those with S-BTcP.

The results of participants' psychosocial assessments are summarized in Table 4. Psychological distress occurred more frequently in those with S-BTcP, but only the difference for the “are you anxious” question was statistically significant (*p*=0.028).

### 3.4. Cancer Pain Data and Psychosocial Assessments in the S-IcP Group

Participants' CP characteristics according to S-IcP status are summarized in Table 5. S-IcP was more often associated with a NPC (43.1% vs 22.1%; *p* < 0.001) and bone involvement (45.9% vs 34.7%; *p*=0.029) and less often with visceral pain (25.4% vs 34.7%; *p*=0.051) and S-BTcP (32% vs 43.2%; *p*=0.027). There were no notable differences in pain location or duration. Differences in pain severity and pain relief measures mirrored those identified in relation to the presence of S-BTcP and were highly statistically significant (*p* < 0.0001). Sleep interference was greater (5.1 ± 1.7 vs 4.7 ± 2.1; *p*=0.087) in those with S-IcP.

Participants' cancer pain management with opioids and adjuvant analgesics according to S-IcP status is summarized in Table 6. Similar to S-BTcP, opioid doses were higher in the presence of S-IcP (log_*n*_MEDD: 3.6 ± 1.0 vs 3.0 ± 1.6, *p* < 0.0001) and those with S-IcP were more likely to be prescribed an opioid. Adjuvant analgesic use was higher (62.4% vs 51.1%; *p*=0.027) when S-IcP was present, reflecting higher corticosteroid, antiepileptic, and bisphosphonate use. Participants' psychosocial assessment results are summarized in Table 7. A substance abuse history occurred more frequently (28.7% vs 17.9%; *p*=0.013) in those with S-IcP.

### 3.5. Subgroup Comparisons in Relation to Clinicodemographic Variables

In comparison of the S-BTcP-only (*n* = 82) and S-IcP-only (*n* = 123) subgroups, the S-IcP-only subgroup had more frequent exposure to recent radiotherapy (61% vs 42.7%; *p*=0.010), chemotherapy (52% vs 37.8%; *p*=0.045), \bisphosphonate treatment (5.7% vs 0%; *p*=0.028), greater mean sleep interference (5.23 vs 4.65; *p*=0.028), and higher frequency of brain metastases (9.8% vs 2.4%; *p*=0.042). The S-BTcP-only subgroup had a comparatively higher proportion of participants (64.6% vs 47.2%; *p*=0.014) with soft tissue involvement. In comparing the subgroup with both S-BTcP and S-IcP (*n* = 58) with either of the S-BTcP-only or S-IcP-only subgroups, there were no statistically significant differences in BPI pain intensity or pain interference items.

### 3.6. Strength of Association of Clinicodemographic Variables with S-BTcP and S-IcP

Logistic regression analyses examining the association of clinicodemographic variables with the presence of S-BTcP (*N* = 140) as the dependent variable, are summarized in Table 1S. Eighteen independent variables were entered into the initial multivariable model; 12 were retained in the final model, in which 6 had statistically significant (*p* < 0.05) associations with the presence of S-BTcP: head and neck location of pain (OR: 2.53; 95% CI: 1.20–5.37), NPC (OR: 2.39; 95% CI: 1.34–4.26), and BPI average pain intensity (OR: 1.64; 95% CI: 1.36–1.99) were positively associated; abdominal location of pain (OR: 0.324; 95% CI: 0.120–0.873), presence of S-IcP (OR: 0.207; 95% CI: 0.116–0.369), and PMI-negative status (OR: 0.443; 95% CI: 0.213–0.918) were negatively associated. In Hosmer–Lemeshow testing, this model had a chi-square value of 9.05 and a corresponding *p* value of 0.34, indicating a good fit. The model's discriminative ability was good, as reflected by an AUC of 0.820.

The logistic regression analyses with S-IcP (*N* = 181) as the dependent variable are summarized in Table 2S. Twenty independent variables were entered into the initial multivariable model and 11 were retained in the final model, in which 7 had statistically significant (*p* < 0.05) associations with the presence of S-IcP: recent radiotherapy treatment (OR: 1.7; 95% CI: 1.02–2.83), NPC (OR: 2.06; 95% CI: 1.17–3.60), soft tissue pain component (OR: 2.36; 95% CI: 1.3–4.3), BPI average pain intensity (OR: 1.83; 95% CI: 1.51–2.22) and BPI sleep interference (OR: 1.14; 95% CI: 1.0–1.3) were positively associated; presence of S-BTcP (OR: 0.177; 95% CI: 0.096–0.328) and having PMI-negative status (OR: 0.267; 95% CI: 0.132–0.541) were negatively associated. In Hosmer-Lemeshow testing, this model had a Chi Square value of 5.93 and a corresponding *p* value of 0.66, indicating a good fit. The model's discriminative ability was good, based on an AUC of 0.806.

## 4. Discussion

Based on the 7-day occurrence of transitory pain flaring and a reported BPI-worst or BPI-now pain intensity in the moderate to severe (≥4) range, this study's prevalence estimates of S-BTcP and S-IcP were 37.7% and 48.8%, respectively. These are consistent with corresponding estimates of 41% and 44% reported with more stringent diagnostic criteria [13] and the pooled systematic review prevalence of 39.9% in CP clinics [14]. The prevalence of IcP in patients receiving palliative care was 28%–31% in regional study samples and 48% in an international study [23, 24, 39], using the ECS-CP criteria for IcP. Our study prevalence estimates are relatively liberal, as apart from applying the BPI score criteria, the episodic pain types were reported regardless of either the background pain control status or of opioid use. However, over 95% of those in the S-BTcP and S-IcP groups were using opioid analgesics. Although we previously reported opioid undertreatment in this study sample [40], the PMI status of those with S-BTcP or S-IcP did not suggest undertreatment in these specific groups, but rather reflected a propensity for higher opioid dosing. Although the designation of episodic pain status was possible for all participants, inability to do this has been reported in 17% of participants in a recent study using the ECS-CP [39]. Collectively, these data raise issues regarding the taxonomy of episodic BTcP and IcP.

The categorical designation of mild pain is not consistent with that in the literature: [41, 42] some use 0–3 for this category [43], whereas others use 0–4 [41]. Survey data suggest the cutoff score for controlled background pain used in defining BTcP is a major source of variability in published prevalence estimates of BTcP [16, 44]. Use of a cutoff of ≤6 and a standardized assessment tool in an Italian multicentre study yielded a BTcP prevalence of 60% [16], whereas a cutoff of ≤4 in an international study yielded a BTcP prevalence of 19.8% for the past week [44]. In the latter study, 43.4% of patients reported pain flares in the preceding 24 hours. In the current study, higher prevalence estimates of 42.9% and 59.3% for BTcP and IcP occurred when based solely on patient history. This probably reflects an open approach to the patient narrative on their pain history phenomena rather than adopting a more closed approach that is driven by the need to categorize pain as being specifically breakthrough or incident in nature.

Given the need for patient input and patient reported outcomes [45, 46], such as the attainment of personalised pain goal [47], perception of BTcP control [48], and use of rescue medication for BTcP [49], the patient reports of transitory pain flares or exacerbations warrant assessment and evaluation particularly in terms of distress, whatever the cutoff used or not used for controlled baseline pain. This pragmatic approach aligns with the EAPC Delphi survey of BTcP researchers, which concluded that “all significant transient pain exacerbations” could be included under the broad term, episodic pain [18], although linguistic controversy persists [21]. However, use of more stringent definitions and standardized algorithms for BTcP and IcP may be indicated in trials examining therapeutic intervention for these pains and in cohort studies examining their associations and risk factors.

Previous studies have demonstrated associations between BTcP and greater psychological distress [50] and functional interference [13, 22]. The presence of S-BTcP and S-IcP was both associated with higher pain intensity measures, NPC, and higher opioid dosing in this study, consistent with other studies [17, 22, 51]. However, the BPI interference scores and the HADS and ET scores surprisingly did not differ between groups, except for an independent association between sleep interference and S-IcP. The uniformly high level of psychological distress in the entire study sample was notable and perhaps differences in relation to S-BTcP or S-IcP were consequently more difficult to detect. The presence of bone metastases, identified in association with BTcP and IcP in other studies [17, 22, 51], was not associated with either of these pains in this study. However, a soft tissue pain component was independently associated with S-IcP. Although an association between BTcP and poorer functional performance has been reported [13], it was not identified in this study. Both S-BTcP and S-IcP were each negatively predictive of the others presence, possibly reflecting some mutual exclusiveness in the operationalization of the study-defined criteria for the episodic pain types.

Although this study used a comprehensive and standardized approach to CP assessment, it has several limitations. First, this was a single-centre study with potential local referral biases, arguably reflected by a relatively high referral of head and neck cancers and low referral of lung cancers. Second, although our study definitional criteria were standardized, they were less stringent than those of the previous studies [11, 13]. Third, although general opioid use was higher in relation to S-BTcP and S-IcP, no data were captured regarding compliance with recommendations for rescue opioid use. Fourth, no data were collected on the frequency or duration of S-BTcPs and S-IcPs and number of pains, other than the fact that they differed from regular background pain when present. Consequently, the BPI-worst score might not have directly coincided with the patient's S-BTcP or S-IcP flare, and contrary to the PMI findings, it could have been related to end-of-dose failure and inadequate pain management. Fifth, this is a delayed report; although it is possible that pain management could have changed since the data were collected, opioid formulations and adjuvant analgesics have not changed substantially in the interim in Portugal.

Clinical practitioners need to recognize that episodic CP in its broadest sense occurs in most patients and warrants evaluation in terms of its significant contribution to the pain presentation and its associated distress. For clinical researchers, this study highlights the need to be aware of the heterogeneity and complexity of episodic CP; further studies using diagnostic algorithms and validated tools are needed to inform the development of valid classification criteria for BTcP and IcP.

## 5. Conclusion

Both BTcP and IcP occur frequently among CP clinic patients, but their varying definitions hinder prevalence comparisons. Patients frequently report transient pain flares that may not fit the conventional criteria for BTcP and IcP but could be included under the umbrella term of episodic pain. Both BTcP and IcP are independently associated with a neuropathic pain component, higher pain intensity, and opioid dosing. Furthermore, positive independent associations were noted for head and neck pain location with BTcP, and recent radiotherapy and soft tissue involvement with IcP; negative independent associations occurred with abdominal pain location, co-existing IcP in the case of BTcP, and co-existing BTcP in the case of IcP. Further multicentre studies are required to better characterize the complex phenomena of episodic CP, BTcP, and IcP and thus inform the development of valid classification criteria in their regard.

## Figures and Tables

**Table 1:** Demographic and clinical characteristics of study sample according to study-defined episodic pain categories.

Characteristic	All patients	Study-defined episodic cancer pain categories^a^					
		Breakthrough pain			Incident pain		
	Total *n* = 371 (%)	Absent *n* = 231 (%)	Present *n* = 140 (%)	*p* value	Absent *n* = 190 (%)	Present *n* = 181 (%)	*p* value
Age, mean yrs ± SD	62.1 ± 14.3	62.5 ± 13.6	61.5 ± 15.5	0.494	62.6 ± 14.6	61.7 ± 14.1	0.534
Sex: Female	199 (53.6)	128 (55.4)	71 (50.7)	0.379	104 (54.7)	95 (52.5)	0.664
Primary cancer							
Head and neck	92 (24.8)	44 (19.1)	48 (34.3)	**0.022**	42 (22.1)	50 (27.6)	0.185
Lung	10 (2.7)	5 (2.2)	5 (3.6)		4 (2.1)	6 (3.3)	
Gastrointestinal	82 (22.1)	52 (22.5)	30 (21.4)		44 (23.2)	38 (21.0)	
Breast	43 (11.6)	31 (13.4)	12 (8.6)		22 (11.6)	21 (11.6)	
Genitourinary	79 (21.3)	54 (23.4)	25 (17.9)		36 (19.0)	43 (23.8)	
Others	65 (17.5)	45 (19.5)	20 (14.3)		42 (22.1)	23 (12.7)	
Metastatic sites							
Bone	129 (34.8)	88 (38.1)	41 (29.3)	0.084	63 (33.2)	66 (36.5)	0.504
Lungs	53 (14.3)	35 (15.2)	18 (12.9)	0.540	20 (10.5)	33 (18.2)	**0.034**
CNS	21 (5.7)	16 (6.9)	5 (3.6)	0.175	6 (3.2)	15 (8.3)	**0.033**
Liver	58 (15.6)	45 (19.5)	13 (9.3)	**0.009**	30 (15.8)	28 (15.5)	0.932
Nodal	59 (15.9)	34 (14.7)	25 (17.9)	0.423	32 (16.8)	27 (14.9)	0.612
Soft tissue	47 (12.7)	25 (10.8)	22 (15.7)	0.170	21 (11.1)	26 (14.4)	0.338
Other	106 (32.7)	64 (31.1)	42 (35.6)	0.403	55 (32.5)	51 (32.9)	0.945
Palliative goal	176 (47.4)	97 (42)	79 (56.4)	**0.007**	83 (43.7)	93 (51.4)	0.138
Cognitive deficit	46 (12.4)	28 (12.1)	18 (12.9)	0.835	25 (13.2)	21 (11.6)	0.649
Functional status (ECOG)							
0	120 (32.4)	73 (31.6)	47 (33.6)	0.576	61 (32.1)	59 (32.6)	0.738
1	128 (34.5)	86 (37.2)	42 (30.0)		62 (32.6)	66 (36.5)	
2	61 (16.4)	38 (16.5)	23 (16.4)		36 (19.0)	25 (13.8)	
3	35 (9.4)	19 (8.2)	16 (11.4)		18 (9.5)	17 (9.4)	
4	27 (7.3)	15 (6.5)	12 (8.6)		13 (6.8)	14 (7.7)	
Surgery^b^	112 (30.2)	71 (30.7)	41 (29.3)	0.768	53 (27.9)	59 (32.6)	0.324
Chemotherapy^b^	167 (45.0)	109 (47.2)	58 (41.4)	0.280	76 (40.0)	91 (50.3)	**0.047**
Radiotherapy^b^	176 (47.4)	110 (47.6)	66 (47.1)	0.929	70 (36.8)	106 (58.6)	**<0.001**

ECOG = eastern cooperative oncology group; CNS = central nervous system. ^a^Based on both history and Brief Pain Inventory scores; ^b^Exposure within the last 30 days.

**Table 2:** Cancer pain characteristics of participants in relation to breakthrough pain status.

Characteristic	Breakthrough pain^a^		All patients	*p* value
	Absent *n* = 231 (%)	Present *n* = 140 (%)	Total *n* = 371 (%)	
*Pain mechanisms and topography*				
Neuropathic pain component (NPC)	54 (23.4)	66 (47.1)	120 (32.4)	**<0.001**
*Nociceptive pain*				
Visceral	76 (32.9)	36 (25.7)	112 (30.2)	0.144
Bone	93 (40.3)	56 (40.0)	149 (40.2)	0.961
Soft tissue	108 (46.8)	110 (78.6)	218 (58.8)	**<0.001**
Study-defined incident pain present	123 (53.3)	58 (41.4)	181 (48.8)	**0.027**
*Pain duration*				
≤1 month	36 (15.6)	29 (20.7)	65 (17.5)	0.208
>1 month	195 (84.4)	111 (79.3)	306 (82.5)	
*Principal pain location*				
Upper limb	23 (10.0)	16 (11.4)	39 (10.5)	**<0.001**
Lower limb	33 (14.3)	17 (12.1)	50 (13.5)	
Head and neck	36 (15.6)	54 (38.6)	90 (24.3)	
Thorax	19 (8.2)	9 (6.4)	28 (7.6)	
Breast	4 (1.7)	1 (0.7)	4 (1.4)	
Back	27 (11.7)	13 (9.3)	40 (10.8)	
Abdomen	39 (16.9)	11 (7.9)	50 (13.5)	
Pelvis and perineum	31 (13.4)	16 (11.4)	47 (12.7)	
Multiple sites	19 (8.2)	3 (2.1)	22 (5.9)	
*Brief pain inventory (BPI)*				
Worst pain	6.7 ± 2.9	8.4 ± 1.6	7.4 ± 2.6	**<0.0001**
Least pain	2.6 ± 2.0	3.6 ± 1.5	3.0 ± 1.9	**<0.0001**
Average pain	4.5 ± 2.1	5.6 ± 1.4	4.9 ± 1.9	**<0.0001**
Pain now	4.9 ± 2.8	6.2 ± 1.9	5.4 ± 2.6	**<0.0001**
Pain relief	49.7 ± 29.9	36.4 ± 26.1	44.7 ± 29.2	**<0.0001**
*BPI pain interference*				
General activity	5.5 ± 2.5	5.2 ± 2.7	5.4 ± 2.6	0.179
Mood	7.4 ± 2.3	7.6 ± 2.6	7.5 ± 2.4	0.345
Walking	5.3 ± 2.6	5.3 ± 2.6	5.3 ± 2.6	0.991
Work	4.4 ± 2.8	4.6 ± 3.1	4.5 ± 2.9	0.540
Relations with people	2.9 ± 1.9	3.2 ± 1.9	3.0 ± 1.9	0.075
Sleep	5.0 ± 1.8	4.7 ± 2.1	4.9 ± 1.9	0.097
Enjoyment of life	7.4 ± 2.4	7.4 ± 2.5	7.4 ± 2.4	0.982

^a^Study-defined, based on history and on BPI scores.

**Table 3:** Cancer pain management of participants in relation to their breakthrough pain status.

Characteristic	Breakthrough pain^a^		All patients	*p* value
	Absent *n* = 231 (%)	Present *n* = 140 (%)	Total *n* = 371 (%)	
*Opioid use*				
No regular opioid prescription	39 (16.9)	3 (2.1)	42 (11.3)	**<0.001**
MEDD, median (interquartile range)	30 (15–60)	30 (20–60)	30 (20–60)	0.078
Log_n_ MEDD, mean ± SD	3.1 ± 1.5	3.6 ± 0.9	3.3 ± 1.4	**0.0005**
*Adjuvant analgesic use*				
Corticosteroid	34 (14.7)	27 (19.3)	61 (16.4)	0.250
Benzodiazepine	48 (20.8)	39 (27.9)	87 (23.5)	0.119
Antiepileptic	35 (15.2)	34 (24.3)	69 (18.6)	**0.028**
Antidepressant	34 (14.7)	20 (14.3)	54 (14.6)	0.909
Bisphosphonate	9 (3.9)	2 (1.4)	11 (3.0)	0.174
Use of ≥1 adjuvant	126 (54.6)	84 (60.0)	210 (56.6)	0.304
*Pain Management Index (PMI)*				
PMI negative	59 (25.5)	36 (25.7)	95 (25.6)	0.970

MEDD = total morphine equivalent daily dose in mg, oral. ^a^Study-defined, based on history and on BPI scores.

**Table 4:** Psychosocial assessment of participants in relation to their breakthrough pain status.

Characteristic	Breakthrough pain^a^		All patients	*p* value
	Absent *n* = 231 (%)	Present *n* = 140 (%)	Total *n* = 371 (%)	
History of chronic depression	13 (5.6)	9 (6.4)	22 (5.9)	0.752
History of drug or alcohol abuse	46 (19.9)	40 (28.6)	86 (23.2)	0.055
*Positive single question screening*				
Are you depressed?	160 (69.3)	107 (76.4)	267 (72.0)	0.136
Are you anxious?	148 (64.1)	105 (75.0)	253 (68.2)	**0.028**
*Hospital Anxiety Depression Scale (HADS)*				
HADS-Anxiety score > 7	156 (67.5)	106 (75.7)	262 (70.6)	0.094
HADS-Depression score > 7	167 (72.3)	113 (80.7)	280 (75.5)	0.068
*Emotional thermometer (ET)*				
ET-Distress > 3	123 (53.3)	85 (60.7)	208 (56.1)	0.160
ET-anger > 3	128 (55.4)	88 (62.9)	216 (58.2)	0.159
ET-help desired > 3	129 (55.8)	80 (57.1)	209 (56.3)	0.807

^a^Study-defined, based on history and on BPI scores.

**Table 5:** Cancer pain characteristics of participants according to their incident pain status.

Characteristic	Incident pain^a^		All patients	*p* value
	Absent *n* = 190 (%)	Present *n* = 181 (%)	Total *n* = 371 (%)	
*Pain mechanisms and topography*				
Neuropathic component	42 (22.1)	78 (43.1)	120 (32.4)	**<0.001**
*Nociceptive pain*				
Visceral	66 (34.7)	46 (25.4)	112 (30.2)	**0.051**
Bone	66 (34.7)	83 (45.9)	149 (40.2)	**0.029**
Soft tissue	103 (54.2)	115 (63.5)	218 (58.8)	0.068
Study-defined breakthrough pain present	82 (43.2)	58 (32.0)	140 (37.7)	**0.027**

*Pain duration*				
≤1 month	36 (19.0)	29 (16.0)	65 (17.5)	0.459
>1 month	154 (81.1)	152 (84.0)	306 (82.5)	

*Principal pain location*				
Upper limb	19 (10.0)	20 (11.1)	39 (10.5)	0.233
Lower limb	20 (10.5)	30 (16.6)	50 (13.5)	
Head and neck	44 (23.2)	46 (25.4)	90 (24.3)	
Thorax	16 (8.4)	12 (6.6)	28 (7.6)	
Breast	2 (1.1)	3 (1.7)	5 (1.4)	
Back	18 (9.5)	22 (12.2)	40 (10.8)	
Abdomen	31 (16.3)	19 (10.5)	50 (13.5)	
Pelvis and perineum	24 (12.6)	23 (12.7)	47 (12.7)	
Multiple sites	16 (8.4)	6 (3.3)	22 (5.9)	

*Brief Pain Inventory (BPI)*				
Worst pain	6.4 ± 3.1	8.4 ± 1.4	7.4 ± 2.6	**<0.0001**
Least pain	2.5 ± 2.1	3.5 ± 1.5	3.0 ± 1.9	**<0.0001**
Average pain	4.4 ± 2.2	5.5 ± 1.3	4.9 ± 1.9	**<0.0001**
Pain now	4.5 ± 3.0	6.3 ± 1.8	5.4 ± 2.6	**<0.0001**
Pain relief	53.7 ± 32.1	35.2 ± 22.2	44.7 ± 29.2	**<0.0001**

*BPI pain interference*				
General activity	5.4 ± 2.6	5.4 ± 2.6	5.4 ± 2.6	0.959
Mood	7.5 ± 2.3	7.5 ± 2.6	7.5 ± 2.4	0.885
Walking	5.3 ± 2.7	5.4 ± 2.5	5.3 ± 2.6	0.734
Work	4.3 ± 2.8	4.8 ± 3.0	4.5 ± 2.9	0.127
Relations with people	2.9 ± 1.8	3.0 ± 2.0	3.0 ± 1.9	0.585
Sleep	4.7 ± 2.1	5.1 ± 1.7	4.9 ± 1.9	0.087
Enjoyment of life	7.6 ± 2.4	7.3 ± 2.5	7.4 ± 2.4	0.176

^a^Study-defined, based on history and on BPI scores.

**Table 6:** Cancer pain management of participants according to their incident pain status.

Characteristic	Incident pain^a^		All patients	*p* value
	Absent *n* = 190 (%)	Present *n* = 181 (%)	Total *n* = 371 (%)	
*Opioid use*				
No regular opioid prescription	36 (19.0)	6 (3.3)	42 (11.3)	**<0.001**
MEDD, median (interquartile range)	30 (13.5–60)	30 (22.5–60)	30 (20–60)	**0.003**
Log_*n*_ MEDD, mean ± SD	3.0 ± 1.6	3.6 ± 1.0	3.3 ± 1.4	**<0.0001**

*Adjuvant analgesic use*				
Corticosteroid	21 (11.1)	40 (22.1)	61 (16.4)	**0.004**
Benzodiazepine	45 (23.7)	42 (23.2)	87 (23.5)	0.913
Antiepileptic	23 (12.1)	46 (25.4)	69 (18.6)	**0.001**
Antidepressant	30 (15.8)	24 (13.3)	54 (14.6)	0.490
Bisphosphonate	2 (1.1)	9 (5.0)	11 (3.0)	**0.026**
Use of ≥1 adjuvant	97 (51.1)	113 (62.4)	210 (56.6)	**0.027**

*Pain Management Index (PMI)*				
PMI negative	55 (29.0)	40 (22.1)	95 (25.6)	0.131

MEDD = total morphine equivalent daily dose in mg, oral. ^a^Study-defined, based on history and on BPI scores.

**Table 7:** Psychosocial assessments of participants according to their incident pain status.

Characteristic	Incident pain^a^		All patients	*p* value
	Absent *n* = 190 (%)	Present *n* = 181 (%)	Total *n* = 371 (%)	
History of chronic depression	14 (7.4)	8 (4.4)	22 (5.9)	0.229
History of drug or alcohol abuse	34 (17.9)	52 (28.7)	86 (23.2)	**0.013**
*Positive single question screening*				
Are you depressed?	136 (71.6)	131 (72.4)	267 (72.0)	0.864
Are you anxious?	132 (69.5)	121 (66.9)	253 (68.2)	0.588
*Hospital Anxiety Depression Scale (HADS)*				
HADS-Anxiety score > 7	136 (71.6)	126 (69.6)	262 (70.6)	0.678
HADS-Depression score > 7	143 (75.3)	137 (75.7)	280 (75.5)	0.924
*Emotional thermometer (ET)*				
ET-Distress > 3	100 (52.6)	108 (59.7)	208 (56.1)	0.172
ET-anger > 3	113 (59.5)	103 (56.9)	216 (58.2)	0.616
ET-help desired > 3	101 (53.2)	108 (59.7)	209 (56.3)	0.206

^a^Study-defined, based on history and on BPI scores.

## Data Availability

The data used to support the findings of this study are included within the article. Further details are available from the corresponding author upon request.

## Supplementary Materials

Supplementary Materials Table 1S: logistic regression examination of strength of association of variables with study-defined episodic breakthrough pain. Table 2S: logistic regression examination of strength of association of variables with study-defined episodic incident pain.
