# Supplementary material for: Episodic Cancer Pain: Patient Reporting, Prevalence, and Clinicodemographic Associations at Initial Cancer Pain Clinic Assessment
Source: Pain Res Manag. 2020 May 22;2020:6190862. doi: 10.1155/2020/6190862 (PMC7261329; doi:10.1155/2020/6190862)
Supplement: Supplementary Materials — Table 1S: logistic regression examination of strength of association of variables with study-defined episodic breakthrough pain. Table 2S: logistic regression examination of strength of association of variables with study-defined episodic incident pain. [file 6190862.f1.docx]

**Supplementary File with Table 1S and Table 2S**

**Table 1S Logistic regression examination of strength of association of variables with study-defined episodic breakthrough pain**

| **Variables assessed at 1^st^ clinic visit** | **Bivariable OR (95% CI)** | | **P value** |  | **Multivariable OR (95% CI)^a^** | | **P value** |
| --- | --- | --- | --- | --- | --- | --- | --- |
| **Sex** |  | |  |  |  | |  |
| **Male** | **Ref** | |  |  | **Ref** | |  |
| **Female** | **0.828** | **(0.544-1.26)** | **0.379** |  | **1.43** | **(0.815-2.52)** | **0.211** |
| **Metastatic disease sites** |  |  |  |  |  |  |  |
| **Bone metastases** | **0.673** | **(0.429-1.06)** | **0.085** |  | **0.550** | **(0.302-1.00)** | **0.051** |
| **Liver metastases** | **0.423** | **(0.219-0.816)** | **0.010** |  | **0.574** | **(0.254-1.30)** | **0.182** |
| **Palliative treatment goal** | **1.79** | **(1.17-2.73)** | **0.007** |  | **1.73** | **(0.980-3.05)** | **0.059** |
| **Pain location** |  | |  |  |  | |  |
| **Upper or Lower Limbs** | **Ref** | |  |  | **Ref** | |  |
| **Head & Neck** | **2.55** | **(1.39-4.65)** | **0.002** |  | **2.53** | **(1.20-5.37)** | **0.015** |
| **Thorax, Breast or Back** | **0.781** | **(0.406-1.50)** | **0.459** |  | **0.730** | **(0.335-1.59)** | **0.427** |
| **Abdomen** | **0.479** | **(0.216-1.06)** | **0.069** |  | **0.324** | **(0.120-0.873)** | **0.026** |
| **Pelvis & Perineum** | **0.876** | **(0.418-1.84)** | **0.726** |  | **0.554** | **(0.228-1.35)** | **0.193** |
| **Multiple Sites** | **0.268** | **(0.074-0.975)** | **0.046** |  | **0.372** | **(0.086-1.62)** | **0.187** |
| **Pain mechanism** |  |  |  |  |  |  |  |
| **Neuropathic component present** | **2.92** | **(1.86-4.59)** | **<0.001** |  | **2.39** | **(1.34-4.26)** | **0.003** |
| **Study-defined incident pain status** |  |  |  |  |  |  |  |
| **Incident pain present** | **0.621** | **(0.406-0.949)** | **0.028** |  | **0.207** | **(0.116-0.369)** | **<0.001** |
| **Pain intensity** |  |  |  |  |  |  |  |
| **BPI Pain Average Rating** | **1.38** | **(1.22-1.56)** | **<0.001** |  | **1.64** | **(1.36-1.99)** | **<0.001** |
| **BPI Pain interference** |  |  |  |  |  |  |  |
| **Relationships with people** | **1.11** | **(0.989-1.24)** | **0.076** |  | **1.13** | **(0.981-1.29)** | **0.091** |
| **Psychological distress** |  |  |  |  |  |  |  |
| **HADS Anxiety ≤ 7** | **Ref** | |  |  | **Ref** | |  |
| **HADS Anxiety > 7** | **1.50** | **(0.933-2.41)** | **0.095** |  | **1.57** | **(0.869-2.82)** | **0.135** |
| **ET Distress score < 4** | **Ref** | |  |  | **Ref** | |  |
| **ET Distress score ≥ 4** | **1.36** | **(0.886-2.08)** | **0.161** |  | **1.65** | **(0.973-2.79)** | **0.063** |
| **Pain management index (PMI)** |  |  |  |  |  |  |  |
| **PMI negative status** | **1.01** | **(0.624-1.63)** | **0.970** |  | **0.443** | **(0.213-0.918)** | **0.028** |
| **Age** | **0.995** | **(0.980-1.01)** | **0.493** |  | **---** |  |  |
| **Functional status** |  |  |  |  |  |  |  |
| **ECOG (0-2)** | **Ref** | |  |  |  |  |  |
| **ECOG (3-4)** | **1.45** | **(0.835-2.51)** | **0.188** |  | **---** |  |  |
| **Soft tissue metastases** | **1.54** | **(0.830-2.84)** | **0.172** |  | **---** |  |  |
| **BPI sleep interference** | **0.911** | **(0.816-1.02)** | **0.097** |  | **---** |  |  |
| **History of drug or alcohol abuse** | **1.61** | **(0.987-2.62)** | **0.057** |  | **---** |  |  |
| **Depression screen** |  |  |  |  |  |  |  |
| **HADS Depression < 7** | **Ref** | |  |  |  |  |  |
| **HADS Depression ≥ 7** | **1.60** | **(0.964-2.67)** | **0.069** |  | **---** |  |  |

**OR = Odds Ratio; BPI = Brief Pain Inventory; HADS = Hospital Anxiety Depression Scale; ET = emotion thermometer; ECOG = Eastern Cooperative Oncology Group.**

**^a^ Multivariable model’s goodness of fit: Hosmer-Lemeshow Chi Sq = 9.05 (p = 0.34); model’s discrimination: area under the receiver operating characteristic curve = 0.820.**

**Table 2S Logistic regression examination of strength of association of variables with study-defined episodic incident pain**

| **Variables assessed at 1^st^ clinic visit** | **Bivariable OR (95% CI)** | | **P value** |  | **Multivariable OR (95% CI)^a^** | | **P value** |
| --- | --- | --- | --- | --- | --- | --- | --- |
| **Cancer diagnosis** |  | |  |  |  | |  |
| **Lung or Other** | **Ref** | |  |  |  | |  |
| **Gastrointestinal** | **1.37** | **(0.725-2.59)** | **0.332** |  | **1.74** | **(0.733-4.13)** | **0.209** |
| **Breast** | **1.51** | **(0.710-3.23)** | **0.283** |  | **1.36** | **(0.528-3.52)** | **0.521** |
| **Genitourinary** | **1.90** | **(0.997-3.60)** | **0.051** |  | **1.73** | **(0.730-4.08)** | **0.214** |
| **Head & Neck** | **1.89** | **(1.02-3.51)** | **0.044** |  | **1.15** | **(0.440-3.03)** | **0.772** |
| **Metastatic disease sites** |  |  |  |  |  |  |  |
| **Lung metastases** | **1.90** | **(1.04-3.45)** | **0.036** |  | **1.89** | **(0.906-3.92)** | **0.090** |
| **Oncological treatment** |  |  |  |  |  |  |  |
| **Radiotherapy^b^** | **2.42** | **(1.60-3.68)** | **<0.001** |  | **1.70** | **(1.02-2.83)** | **0.043** |
| **Principal pain location** |  |  |  |  |  |  |  |
| **Thorax, Breast or Back** | **Ref** | |  |  | **Ref** | |  |
| **Head & Neck** | **1.02** | **(0.549-1.89)** | **0.957** |  | **1.76** | **0.625-4.98)** | **0.284** |
| **Upper or Lower Limbs** | **1.25** | **(0.670-2.32)** | **0.485** |  | **1.97** | **(0.920-4.23)** | **0.081** |
| **Abdomen** | **0.596** | **(0.287-1.24)** | **0.167** |  | **0.773** | **(0.291-2.06)** | **0.606** |
| **Pelvis & Perineum** | **0.932** | **(0.448-1.94)** | **0.852** |  | **0.899** | **(0.338-2.39)** | **0.830** |
| **Multiple Sites** | **0.365** | **(0.128-1.04)** | **0.058** |  | **0.577** | **(0.150-2.22)** | **0.423** |
| **Pain mechanism** |  |  |  |  |  |  |  |
| **Neuropathic component present** | **2.67** | **(1.70-4.19)** | **<0.001** |  | **2.06** | **(1.17-3.60)** | **0.012** |
| **Pain topography** |  |  |  |  |  |  |  |
| **Soft tissue element** | **1.47** | **(0.971-2.23)** | **0.069** |  | **2.36** | **(1.30-4.30)** | **0.005** |
| **Study-defined BTP status** |  |  |  |  |  |  |  |
| **BTP pain (no trigger) present** | **0.621** | **(0.406-0.949)** | **0.028** |  | **0.177** | **(0.096-0.328)** | **<0.001** |
| **Pain intensity** |  |  |  |  |  |  |  |
| **BPI Pain Average Rating** | **1.42** | **(1.26-1.61)** | **<0.001** |  | **1.83** | **(1.51-2.22)** | **<0.001** |
| **Pain interference** |  |  |  |  |  |  |  |
| **BPI Sleep** | **1.10** | **(0.986-1.22)** | **0.087** |  | **1.14** | **(1.00-1.30)** | **0.049** |
| **Psychological distress** |  |  |  |  |  |  |  |
| **ET Distress < 4** | **Ref** | |  |  |  |  |  |
| **ET Distress ≥ 4** | **1.33** | **(0.882-2.01)** | **0.173** |  | **1.47** | **(0.891-2.41)** | **0.132** |
| **Pain management index (PMI)** |  |  |  |  |  |  |  |
| **PMI negative status** | **0.70** | **(0.435-1.12)** | **0.132** |  | **0.267** | **(0.132-0.541)** | **<0.001** |
| **Age** | **0.996** | **(0.981-1.01)** | **0.533** |  | **---** |  |  |
| **Sex** |  | |  |  |  | |  |
| **Male** | **Ref** | |  |  |  | |  |
| **Female** | **0.914** | **(0.607-1.37)** | **0.664** |  | **---** |  |  |
| **Functional status** |  |  |  |  |  |  |  |
| **ECOG (0-2)** | **Ref** | |  |  |  |  |  |
| **ECOG (3-4)** | **1.06** | **(0.614-1.83)** | **0.834** |  | **---** |  |  |
| **Palliative treatment goal** | **1.36** | **(0.905-2.05)** | **0.138** |  | **---** |  |  |
| **Oncological management** |  |  |  |  |  |  |  |
| **Surgery^b^** | **1.25** | **(0.802-1.95)** | **0.324** |  | **---** |  |  |
| **Chemotherapy^b^** | **1.52** | **(1.01-2.29)** | **0.047** |  | **---** |  |  |
| **Pain topography** |  |  |  |  |  |  |  |
| **Bone** | **1.59** | **(1.05-2.42)** | **0.029** |  | **---** |  |  |
| **Visceral** | **0.640** | **(0.409-1.00)** | **0.051** |  | **---** |  |  |
| **History of drug or alcohol abuse** | **1.85** | **(1.13-3.02)** | **0.014** |  | **---** |  |  |

**OR = Odds Ratio; BTP = breakthrough pain; BPI = Brief Pain Inventory; ET = emotion thermometer; ECOG = Eastern Cooperative Oncology Group.**

**^a^ Multivariable model’s goodness of fit: Hosmer-Lemeshow Chi Sq = 5.93 (p = 0.66); model’s discrimination: area under the receiver operating characteristic curve = 0.806.**

**^b^ Within the last 30 days**
